# Genomic analysis of group B *Streptococcus* from milk demonstrates the need for improved biosecurity: a cross-sectional study of pastoralist camels in Kenya

**DOI:** 10.1186/s12866-021-02228-9

**Published:** 2021-07-19

**Authors:** Dinah Seligsohn, Chiara Crestani, Taya L. Forde, Erika Chenais, Ruth N. Zadoks

**Affiliations:** 1 Department of Animal Health and Antimicrobial Strategies, National Veterinary Institute, SE- 75189 Uppsala, Sweden; 2 Department of Clinical Sciences, Swedish University of Agricultural Sciences, Uppsala, Sweden; 3 Institute of Biodiversity, Animal Health and Comparative Medicine, University of Glasgow, Glasgow, United Kingdom; 4 Department of Disease Control and Epidemiology, National Veterinary Institute, SE-75189 Uppsala, Sweden; 5 Sydney School of Veterinary Science, University of Sydney, Sydney, Australia

**Keywords:** *Streptococcus agalactiae*, Nomadic, Dairy, Camelids, Molecular epidemiology, Intramammary infection, Biosecurity

## Abstract

**Background:**

*Streptococcus agalactiae* (Group B *Streptococcus*, (GBS)) is the leading cause of mastitis (inflammation of the mammary gland) among dairy camels in Sub-Saharan Africa, with negative implications for milk production and quality and animal welfare. Camel milk is often consumed raw and presence of GBS in milk may pose a public health threat. Little is known about the population structure or virulence factors of camel GBS. We investigated the molecular epidemiology of camel GBS and its implications for mastitis control and public health.

**Results:**

Using whole genome sequencing, we analysed 65 camel milk GBS isolates from 19 herds in Isiolo, Kenya. Six sequence types (STs) were identified, mostly belonging to previously described camel-specific STs. One isolate belonged to ST1, a predominantly human-associated lineage, possibly as a result of interspecies transmission. Most (54/65) isolates belonged to ST616, indicative of contagious transmission. Phylogenetic analysis of GBS core genomes showed similar levels of heterogeneity within- and between herds, suggesting ongoing between-herd transmission. The lactose operon, a marker of GBS adaptation to the mammary niche, was found in 75 % of the isolates, and tetracycline resistance gene *tet*(M) in all but two isolates. Only the ST1 isolate harboured virulence genes *scpB* and *lmb*, which are associated with human host adaptation.

**Conclusions:**

GBS in milk from Kenyan camel herds largely belongs to ST616 and shows signatures of adaptation to the udder. The finding of similar levels of within- and between herd heterogeneity of GBS in camel herds, as well as potential human-camel transmission highlights the need for improved internal as well as external biosecurity to curb disease transmission and increase milk production.

**Supplementary Information:**

The online version contains supplementary material available at 10.1186/s12866-021-02228-9.

## Background

In the arid and semi-arid lands of the Horn of Africa, camels are valuable assets for household income and food security, and commonly kept for milk production. In these areas, where desertification and prolonged droughts are constraints for food production, camel pastoralism is an integral part of the sustenance of the inhabitants, and in some areas camel milk and meat contribute to more than 50 % of the diet [1, 2]. Camels are well-adapted to harsh conditions and can continue to produce milk despite limited access to feed and water, which sets them apart from other types of livestock [3]. Kenya has the third largest camel population globally [4]. The majority of these camels are kept by pastoralists, who adhere to nomadic husbandry traditions, browsing camels over large areas and milking by hand [5]. These pastoralists live in close contact with their animals, and milk is consumed without prior pasteurization, both practices that contribute to zoonotic disease transmission risks [2].

Mastitis, inflammation of the mammary gland, is a frequently-occurring problem among dairy camels with negative implications for production and animal welfare [6, 7]. *Streptococcus agalactiae*, or group B *Streptococcus* (GBS), has been identified as a common cause of both clinical mastitis (CM) and subclinical mastitis (SCM) in camels [8, 9], often resulting in chronic infections [10], reduced milk yield [11] and high bacterial counts in milk [12]. In Kenya, a lack of involvement of veterinary services and poor compliance with recommended dosing regimens in camels [13], in combination with the common use of substandard antimicrobial products [14, 15], may serve as drivers in the development of antimicrobial resistance. Resistance has been reported in bacteria from camel milk within the region, including GBS [8, 16, 17].

In the bovine dairy industry, the molecular epidemiology of GBS largely depends on the local context. Studies in dairy herds in northern Europe (Denmark, Finland, Norway) [18–20] and in Australia [21] show limited strain diversity of GBS at farm level, with one genotype predominating in each herd. Differently, in India and Colombia, extensive within-herd heterogeneity has been observed, with an overlap in sequence types (STs) isolated from cattle and people [22, 23]. Variation in access to veterinary services [24], milking hygiene [8], and other biosecurity practices, [25] affecting pathogen transmission and mastitis control may contribute to such differences.

Investigations of the global GBS population have revealed the existence of clonal complexes (CC) associated with specific host species or niches (e.g. the mammary gland) and generalist lineages [22, 26, 27]. Niche adaptation, such as lactose fermentation, has been described in mastitis-causing isolates, and the lactose operon (Lac.2) has been identified as responsible for lactose metabolism in the vast majority of bovine isolates [19, 28, 29]. In addition to genes encoding metabolic properties, virulence genes, including adhesins and invasins, can affect pathogens’ ability to cause infections. For example, *scpB* (C5a peptidase) and *lmb* (laminin-binding protein) have been strongly associated with disease in humans, but not animals [29].

In camels, little is known about the population structure and epidemiology of GBS. Fischer et al. [17] found that isolates from camel mastitis primarily belonged to a common genotype, ST616, but with limited description of spatial or social relations between animals or herds under investigation. Combined molecular and epidemiological investigation is needed to increase the understanding of sources and transmission routes for GBS in pastoralist camel herds, to expand the knowledge base underpinning mastitis control strategies, and to explore the potential threat to public health. Here, we investigate the molecular epidemiology of GBS in dairy camel herds in Kenya using genomic and phylogenetic analysis, as well as spatial mapping.

## Methods

### Study area and selection of herds

This cross-sectional study was undertaken around Isiolo town, located in Isiolo County in central Kenya. Isiolo County is classified as arid or semi-arid with an annual rainfall of approximately 150–250 mm [30]. The camel population in the region was estimated to comprise of 45,309 individuals [31] distributed over 2,050 camel milk producers [32] out of a total human population of 268,002 individuals [33]. Camel keeping households produce milk for household consumption, but there is also an expanding camel milk market with milk being sold commercially along an informal milk value chain.

Herds were selected based on the following inclusion criteria: Pastoralist herd practicing extensive browsing and selling milk, willingness among camel owners to participate, and accessibility of the herds [8]. All selected herds were visited once during the period from February to April 2017, which corresponded to the end of dry season or early wet season. Herd data regarding management was collected digitally on a tablet using free open-source software [34]. All herds belonged to the Mlango-Ngarendare-Burat camel milk cluster [35] and sold milk along the informal milk value chain with end markets in Isiolo town and central Nairobi.

### Milk sampling, bacteriological culture and isolate selection

**Fig. 1:**
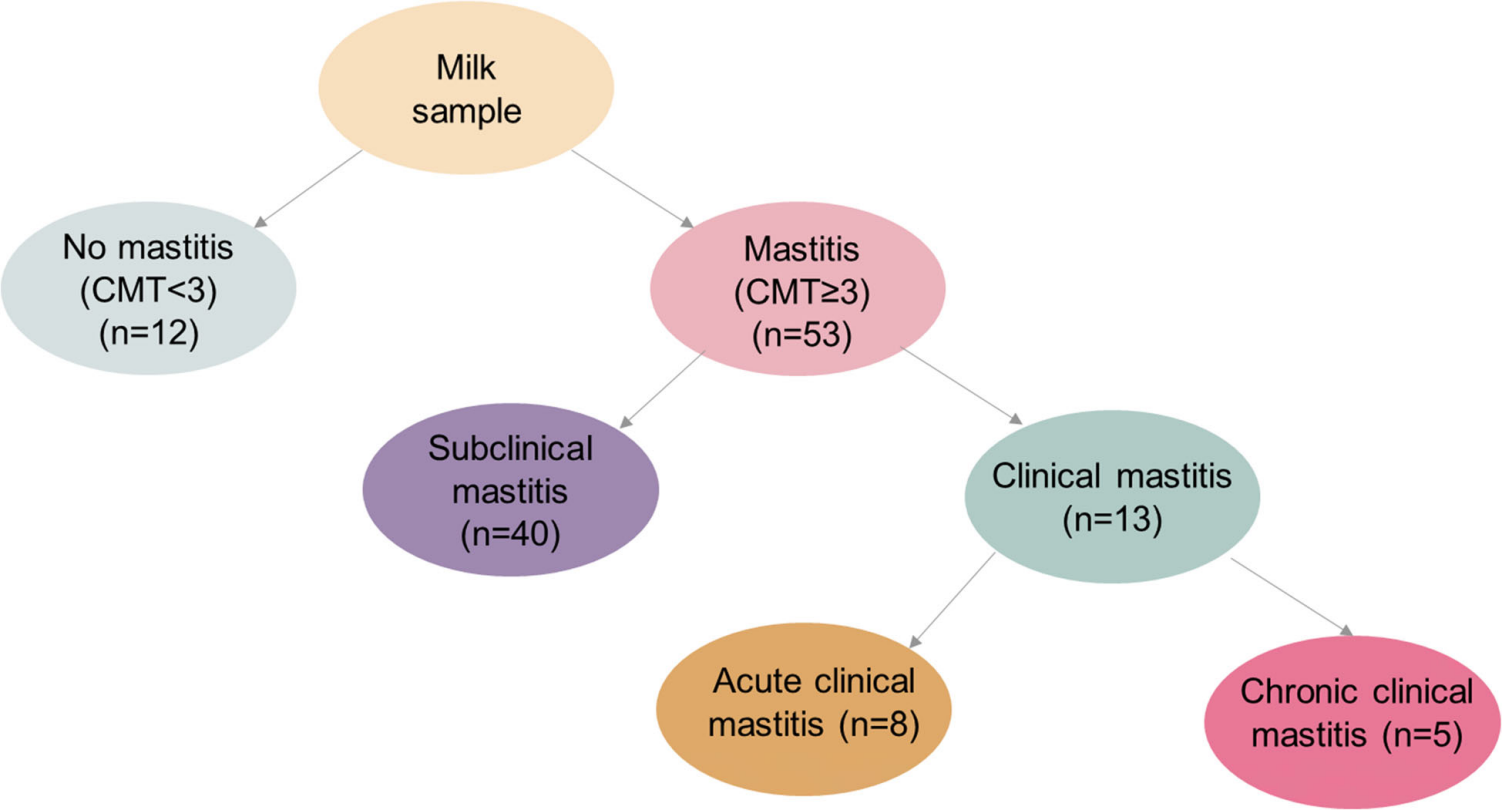
Flowchart of sample and mastitis classification: normal milk (CMT < 3), mastitis (defined as a CMT-score ≥ 3), subclinical mastitis (defined as a CMT- score ≥ 3 and no palpatory or visual changes of the udder or milk), clinical mastitis (defined as CMT-score ≥ 3 and abnormalities in the milk or udder), acute clinical mastitis (ACM) (defined as a CMT-score ≥ 3 and swelling, pain, redness or abnormal milk), and chronic clinical mastitis (CCM) (defined as a CMT-score ≥ 3 and induration of the udder)

Milk was sampled and cultured as described elsewhere [8]. In brief, 20 pastoralist camel herds were visited and a subset of 7 to 13 camels per herd were screened for evidence of mastitis (udder inflammation). Initially, udders were examined clinically and checked for the presence of blind (non-milk producing) quarters and signs of inflammation, such as swelling, increased temperature, pain and redness or palpatory findings of induration of udder tissue. Quarter milk was subjected to the California Mastitis Test (CMT), which provides semi-quantitative measurement of milk leucocyte content as indicator of the degree of inflammation [36]. After discarding the first streaks of milk, approximately 10 mL of milk from each quarter was milked in to the CMT-paddle. Milk was assessed visually for colour, viscosity, presence of blood and clots and then mixed with an equal amount of test reagent. The liquids were mixed by gently rotating the paddle and the viscosity of the solution was scored according to the Scandinavian scoring system (scale from 1 to 5) where 1 represents no change in viscosity, and 5 represents gel formation with a distinct peak [37]. A CMT-score of ≥ 3 was considered indicative of mastitis. For herds with fewer than 20 lactating camels, all animals were sampled. In herds with 20 or more camels every second camel was sampled until a target of 10 camels per herd was reached. Milk samples were frozen at -18 °C to -20 °C for 1 to 7 days prior to culturing. All samples were cultured on blood agar (CM0271, Oxoid, Thermo Fisher Scientific, Waltham, MA) containing 5 % defibrinated sheep blood, and Edwards agar (Oxoid, CM0027) and incubated aerobically at 37℃. After 24 to 48 h, primary species identification was based on colony morphology and catalase testing. Species confirmation was conducted using MALDI-ToF mass spectrometry (MS). Out of 804 quarter milk samples collected across all herds, 154 samples from 65 animals in 19 herds were GBS-positive. One isolate per animal was arbitrarily selected for further analysis. Most (*n* = 53 of 65) isolates were collected from CMT-positive udder quarters, with the remainder (n = 12) collected from CMT-negative quarters. Forty quarters had SCM (CMT-positive but no palpatory or visual changes of the udder or milk) and 13 had signs of CM (visible or palpable abnormalities in the milk or udder). Five CM cases were classified as acute CM (ACM) based on swelling, pain, redness and/or abnormal milk), with the remainder classified as chronic CM (CCM) (based on induration of the udder). Distribution of mastitis categories is shown in Fig. 1. One isolate per camel (1 to 8 isolates per herd, additional file 1) was arbitrarily selected for sequencing and further analysis (additional file 2).

Potential associations between categorical variables were investigated using Pearson’s chi2 test and Fisher’s exact test. A *p*-value of < 0.05 was considered significant. Statistical analyses were performed in Stata (Stata; Stata Statistical Software, release 13.1; StataCorp LP, College Station, TX).

### DNA extraction and sequencing

For DNA extraction, GBS colony material was collected with a calibrated loop (1 µl) and suspended in 600 µl nuclease free water (Sigma-Aldrich, St Louis, MO, USA). The suspension was mixed with 0.1 mm silica beads (BioSpec Products Inc., Bartlesville, USA) and added to the FastPrep24 (MP Biomedicals LLC, Irvine, CA, USA) and then run at 6.5 m/s for three 2-minute cycles. DNA was extracted from 200 µl samples using the IndiMag Pathogen kit (Indical Bioscience GmbH, Leipzig, Germany) and eluted in nuclease free water. DNA concentration was measured using the Invitrogen Qubit 3.0 Fluorometer (ThermoFisher Scientific Inc., Waltham, MA, USA), and adjusted to 7.5 ng/µL. Library preparation and whole genome sequencing were performed by Clinical Genomics, Science for Life Laboratory (Clinical Genomics, Solna, Sweden) on the Illumina NovaSeq (Illumina, Inc. CA, US) resulting in paired end reads of 150 bp in length.

### Antimicrobial susceptibility and virulence testing

Phenotypic antimicrobial susceptibility testing was carried out as previously described [8]. Minimum inhibitory concentrations were determined using broth microdilution. Testing was performed according to the recommendations of the Clinical and Laboratory Standards Institute [38] using VetMIC panels (SVA, Uppsala, Sweden). SRST2 v0.2.0 [39] was used to detect antimicrobial resistance (AMR) genes from raw sequence reads with the ARG-ANNOT v3 database [40].

Lactose fermentation was assessed phenotypically by inoculating each of the selected isolates onto bromocresol purple lactose agar (SVA, Uppsala, Sweden). A yellow colour change of the colonies indicated lactose fermentation whereas purple colonies were classified as negative for lactose fermentation. *Escherichia coli* ATCC35218 and *Proteus mirabilis* CCUG26767 were used as positive and negative controls. Plates were incubated aerobically at 37℃ and checked for colour change at 24, 48 and 72 h. Assembled genomes (see below) were scanned for the presence of the lactose operon (Lac.2) [28] with a BLASTn v2.9.0 search [41] based on a database of four known Lac.2 genotypic variants [27, 42]. Minimum thresholds for identity (ID) and query coverage (QC) were both set at 90 %. Lac.2-negative isolates based on BLAST searches were further scanned for annotations related to Lac.2 in files obtained using Prokka v1.14.6 [43]. To confirm presence/absence of the Lac.2 operon in the chromosome, a PCR targeting a ≈ 2.5-kbp region straddling *lacEG* was used. Positive and negative controls were selected from the study isolates based on genomic detection of Lac.2.

Assembled genomes were scanned for the presence of human-associated virulence genes *scpB* and *lmb* [44] using tBLASTn. Capsular serotyping was conducted *in silico* using a standard method [45].

### Phylogenetic and statistical analysis

Reads were filtered for quality and trimmed with ConDeTri suite v2.3 [46]. *De novo* assembly was performed using SPAdes v3.13.1 [47]. Assembly quality was checked with QUAST v5.0.2 [48] and species identity was confirmed with KmerFinder v3.2 [49]. All assembled genomes passed quality control. Multi locus sequence typing (MLST) was carried out with SRST2 and new alleles were submitted for allele number and ST assignment through pubMLST [50].

A core genome alignment was obtained with Snippy v4.6.0 [51] using ILRI112, an ST617 isolate from a Kenyan camel, as reference genome (accession HF952106). A maximum likelihood tree was inferred with RAxML-NG v0.9.0 [52] under a GTR + G model. A map of herd coordinates was created with ggplot in RStudio, with R (v4.0.). All figures were edited using Inkscape [53]).

Pairwise single nucleotide polymorphism (SNP)-distances between ST616 genomes were calculated using pairsnp v0.2.0 [54] Within-herd pairwise distances and between-herd pairwise distances were plotted using matplotlib v3.3.2 [55].

## Results

### Sequence types and serotypes associated with camel mastitis

All 65 sequenced isolates were confirmed as GBS. Among them, only six STs were identified. These included three novel STs that were single locus variants (SLVs) of STs already described (ST1652, SLV of ST617; ST1653 and ST1654, SLVs of ST616). The vast majority of isolates (*n* = 54) belonged to ST616, which was found in all herds but one, followed by ST1652 (*n* = 6) found in five herds, ST612 (*n* = 2) found in two herds, ST1653 (*n* = 1), and ST1654 (*n* = 1), all belonging to previously described camel-associated clonal complexes. One isolate, however, belonged to a completely unrelated clonal complex (CC1) and was identified as ST1. In most herds (*n* = 12), isolates belonged to a single ST, but multiple STs were detected in the remaining seven herds. In five herds (herds A, B, D, J and N), two STs were present and in two herds (P and R), isolates belonged to three STs.

Four capsular serotypes were detected *in silico* (III to VI). The most common serotype was serotype III (*n* = 56), followed by serotype VI (*n* = 6), serotype IV (*n* = 2) and serotype V (*n* = 1). There was perfect concordance between STs and serotypes, with ST616 and its SLVs ST1653 and ST1654 all belonging to serotype III, ST612 isolates to serotype IV and ST1652 to serotype VI. Serotype III was significantly associated with SCM (36 of 56 serotype III isolates) (Fisher’s exact test; *p =* 0.04).

### Antimicrobial susceptibility and virulence testing

Phenotypic susceptibility testing revealed tetracycline resistance (minimum inhibitory concentration (MIC) above 1 µg/mL) in 63 of 65 GBS isolates (MIC-values for tetracycline shown in Additional file 2, for details on the susceptibility testing, please see [8]). The *tet*(M) gene was found in all phenotypically tetracycline-resistant isolates and was absent from the two isolates that were phenotypically susceptible to tetracycline. Apart from the *tet*(M) gene, no more genes coding for antibiotic resistance were found within the GBS genomes.

Lactose fermentation was detected phenotypically in 75 % (n = 49) of the isolates. PCR-results were in 100 % agreement with the findings of phenotypic lactose fermentation and the presence of a lactose operon in the genomic analysis. There was an association between ST and lactose fermentation (Fisher’s exact test; *p =* 0.002) with the majority of ST616 (*n* = 45) and ST1653 (*n* = 1) being lactose fermenters. Three variants of the lactose operon (Lac.2) were detected, including two known genotypic variants (Lac.2b, *n* = 8; Lac.2d, *n* = 26) and a new variant (*n* = 15) that was named Lac.2e. Lac.2e (length = 9,535 bp) has the same gene arrangement as Lac.2a, with the exception of an additional gene in Lac.2e, a glucokinase (*glk*, length = 951 bp), upstream *lacA*. The Lac.2-variants were located at several insertion sites (Lac.2b, *n* = 2; Lac.2d, *n* = 1; Lac.2e *n* = 2), which largely agreed with their integrase types (additional file 3). Multiple STs were associated with each Lac.2 type and multiple Lac.2 types were detected within STs (Table 1) but there was no association between serotype and lactose operon. For mastitis isolates, i.e. those associated with the presence of an inflammatory response, Lac.2d was overrepresented (25 of 41 mastitis-derived isolates), (Pearson’s chi2-test; *p =* 0.001) and the same association was found for isolates from quarters with SCM (19 of 31 SCM isolates), (Pearson’s chi2-test; *p =* 0.001).

**Table 1:** Distribution of lactose operons across sequence types (STs) in 65 GBS isolates from camel milk

	ST						
**Lactose operon**	**1**	**612**	**616**	**1652**	**1653**	**1654**	**Total**
Negative	1	2	9	3		1	16
Lac.2b			6	1	1		8
Lac.2d			24	2			26
Lac.2e			15				15
**Total**	**1**	**2**	**54**	**6**	**1**	**1**	**65**

The human-associated virulence genes *scpB* and *lmb* were detected in a single genome assembly, which belonged to ST1, as part of a known composite transposon [56]. Two *scpB* variants were found within this mobile element, with *scpB1* (1,071 bp) upstream *scpB2* (2,214 bp), followed by *lmb* (additional file 4).

### Phylogenetic analysis

**Fig. 2:**
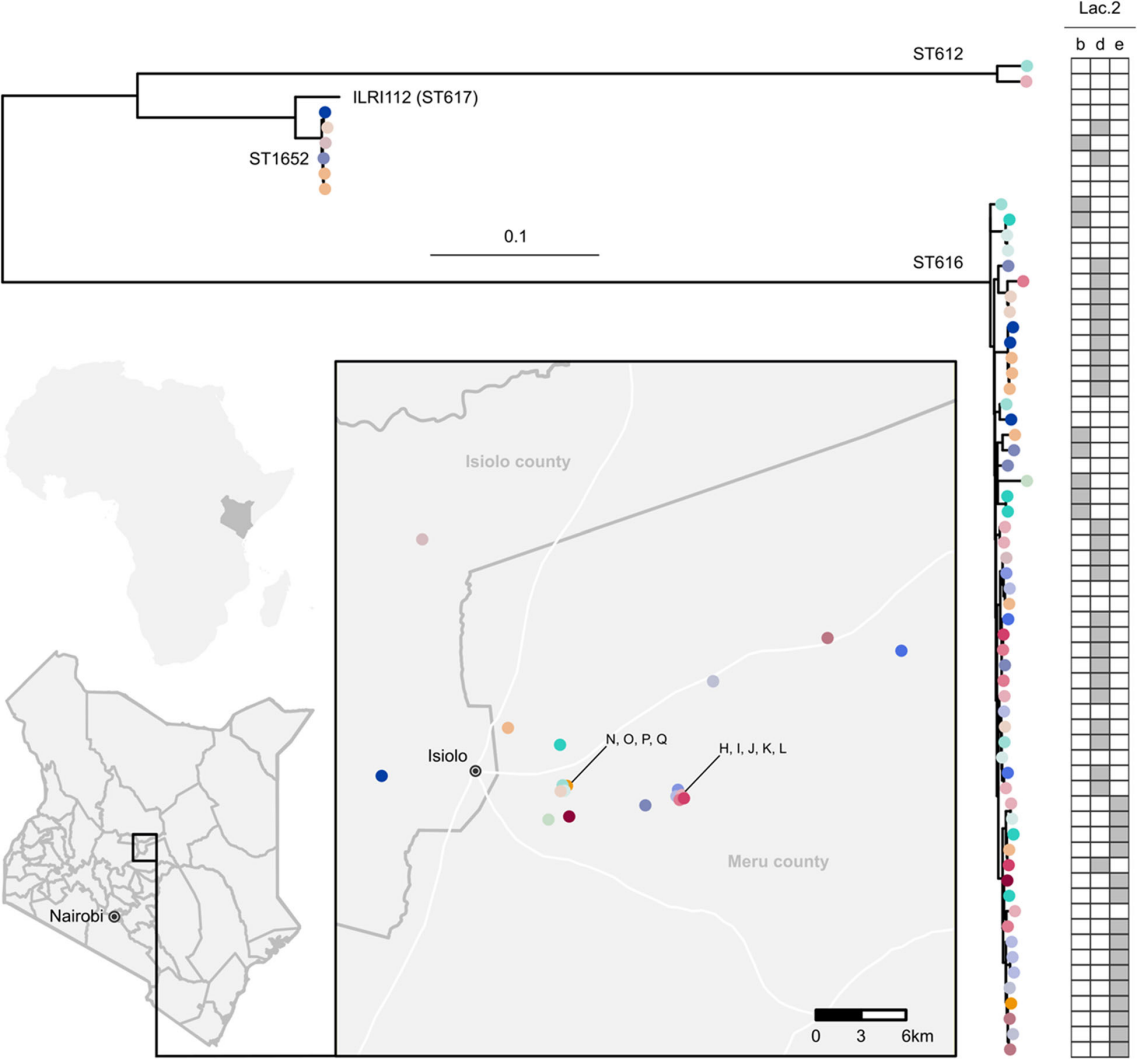
Maximum likelihood phylogenetic tree based on a core genome alignment of 65 camel group B *Streptococcus* (GBS) isolates. Leaf colours correspond to herd of origin (*n* = 19), whose locations are indicated on the map. Sequence types (ST) are shown on the branches, with the ST616 nomenclature also comprising its two single locus variants (ST1653 and ST1654). Grey bars indicate Lac.2 genotypic variants. A single genome assembly belonging to ST1 was removed from the tree to facilitate visualisation. Tree has been rooted at midpoint. ILRI112 (ST617; accession HF952106) was used as reference genome. The map was created with ggplot in RStudio, with R (v4.0.)

After initial inspection, the isolate belonging to ST1 was removed from the phylogenetic tree to better visualise the relatedness among the other isolates. Three main lineages were observed in the core genome phylogenetic tree (Fig. 2). Two lineages corresponded to a single ST (ST612, ST1652), whereas the third and largest lineage included ST616 and its SLVs, ST1653 and ST1654.

**Fig. 3:**
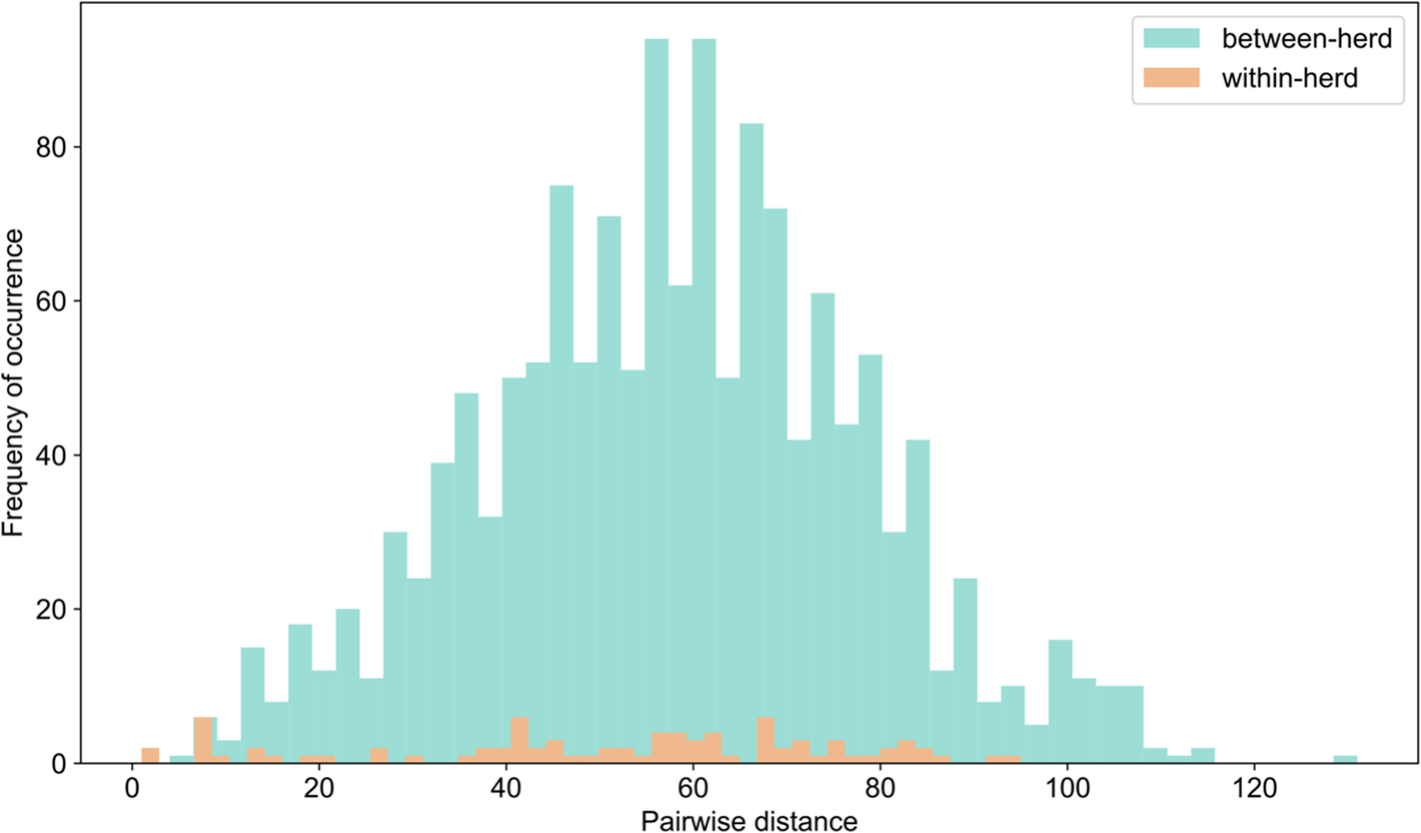
Frequency distribution of pairwise genetic distances calculated between genomes from the largest group B *Streptococcus* lineage from camel milk (sequence types 616, 1653 and 1654) belonging to the same herd (within-herd diversity) or to different herds (between-herd diversity)

Within the major lineage, pairwise genetic distances (i.e. number of single nucleotide polymorphisms) between isolates did not differ for within-herd (mean = 51.33, standard deviation = 24.01) versus between-herd (mean = 57.86, standard deviation = 20.29) comparisons (Fig. 3).

## Discussion

### Genomic analyses show the existence of a predominant niche-adapted strain in camel milk

Here, we describe the genomic diversity of GBS from camel milk in Kenyan dairy herds and demonstrate that the main strain responsible for mastitis shows genetic signatures of adaptation to the mammary gland. ST616 serotype III was the predominant lineage among milk isolates, similar to previous findings [17]. The presence of Lac.2, which always corresponded to phenotypic lactose fermentation in GBS, suggests niche-adaptation to the mammary gland and has been described for GBS [19] as well as for Gram-negative mastitis pathogens [57]. The Lac.2 operon in cattle GBS shows signatures of genetic mobility (e.g. integrase) [28] and several possible insertion sites [42], and in camel GBS, Lac.2 showed similar mobility features. Isolates belonging to STs other than ST616 were less likely to ferment lactose which could indicate infection by strains from extramammary sources, a route of exposure that has also been suggested for dairy cattle [20]. Some non-ST616 isolates encoded Lac.2, which may have been acquired through horizontal gene transfer (HGT) as a means of adaptation to the mammary gland, again as seen in cattle GBS isolates [28]. To further explore infection dynamics within camel herds, within host-diversity needs to be investigated as well as the prevalence and molecular characteristics of extramammary GBS isolates in camels.

Tetracycline resistance was common among the investigated isolates and the *tet*(M) gene was found in all phenotypically resistant isolates. The presence of *tet*(M) has also been described in GBS populations in humans, fish, and, to a much lesser extent, cattle [26, 58, 59]. It has been considered a marker or even driver of expansion of GBS in the human population, starting in the 1940s. Its presence in piscine and bovine isolates has been interpreted as evidence of a human origin of animal GBS [42], but only in isolates from lineages that are shared between host species. Camel GBS is unique and, with the exception of the ST1 isolate reported here, there is no evidence of strain sharing between camels and humans. Considering that tetracycline is commonly used in pastoralist camel herds [13], its presence is likely due to acquisition of mobile genetic elements under selective pressure [17, 60].

### Camel GBS isolates from different herds are genetically related

This is the first report on GBS genomic variation within camel herds. Within-herd heterogeneity of GBS was detected both at ST level (based on ca. 3500 nucleotides from 7 partial housekeeping genes) and within STs (based on single nucleotide polymorphisms detected through whole genome sequencing), with similar heterogeneity within and between herds. Heterogeneity may result from within-herd evolution, as described for GBS in dairy cattle [18]. Considering the finding of GBS in camels with evidence of chronic mastitis such as induration, and the high within-herd prevalence [8], within-camel and within-herd evolution are plausible. In addition, heterogeneity may reflect the occurrence of multiple transmission events between herds. In dairy cattle farms in high income countries, GBS is commonly introduced through the purchase of infected animals [61]. In Kenya, lactating female camels are rarely sold due to their high economic value as milk producers [62]. However, camel dairy herds are not closed, and females are frequently moved between groups depending on reproductive status. Additionally, herds could come into contact with each other during the daytime while browsing or at common watering points. Moreover, herdsmen in commercial herds may move between herds or share duties when camping together, increasing the likelihood of transmission of GBS from one herd to another, especially if not following proper hygiene practices (e.g. washing hands) (personal communication, Yussuf Maalim, Kenya Camel Association). In addition to between-herd biosecurity, within-herd biosecurity is affected by poor hygiene, leading to transmission of GBS between camels. Lack of water and inadequate milking hygiene are common issues in camel pastoralist herds and significant risk factors for mastitis [63, 64]. Fundamental milking hygiene practices, such as cleaning of hands before milking or using gloves, washing of the udder prior to milking or using post-milking teat disinfectant, were lacking in all of the herds included in this study [8]. Some herds would use a milking order, which could be a mastitis-reducing intervention [65], however, this alone is insufficient to halt contagious transmission of GBS without other hygiene measures, which would also require awareness of the existence of SCM, which, by definition, is not visible with the naked eye. Suckling calves may cause contamination of the camel udder [62] but they are unlikely to contribute to camel-to-camel transmission because cross-suckling is thought to be uncommon among camels [66].

### Potential interspecies transmission

This is the first reported case of GBS ST1 isolated from a camel. ST1 is commonly isolated from humans, including healthy carriers, although there are no reports on distribution of human carriage strains in Kenya [67, 68]. This observation may represent interspecies transmission as also observed for cattle [19, 22, 27] and fish [26]. Contamination of the milk sample with a human GBS carriage isolate during the sampling procedure cannot be ruled out, although the isolate was collected from an udder quarter with signs of mastitis (CMT4) and using aseptic sampling technique, suggesting that the bacteria was present intramammarily. The fact that the *scpB1, scpB2* and *lmb-*genes were found only in the ST1 isolate further points towards this isolate being of human origin, as those genes are specifically associated with the human host [29]. The possibility of interspecies transmission could interfere with camel mastitis control strategies as well as pose a risk to the consumers of camel milk. The camel milk industry in Kenya is currently undergoing rapid changes [69] and urban herds kept under more intensive management are becoming more common. In the aquaculture sector, intensification has been associated with the emergence of zoonotic GBS [26] and this risk may also exist in other production animal industries. The direct exposure to GBS of people consuming unpasteurized camel milk warrants further investigation into the potential human health hazard of camel GBS. To investigate potential interspecies transmission, isolates should be collected and compared from camels and from humans living in close contact with camels.

## Conclusions

We found that GBS isolated from camel milk collected in and around Isiolo town (Isiolo County, Kenya) belonged to one predominant sequence type, ST616 or its single locus variants, of which a large proportion showed signs of niche adaptation. In light of this, it is likely that mastitis-causing GBS strains in camels are largely udder-bound and this demonstrates the need for improved milking hygiene. The similarity in heterogeneity of isolates within and across herds suggests that internal and external biosecurity measures would be needed to reduce within- and between herd-transmission, respectively. Our finding of one likely human-derived isolate (ST1) in an infected udder strongly suggests that human to camel transmission is possible, and this potential risk should be further explored. Biosecurity is a cornerstone of disease control; in order for it to be feasible, sustainable and thus efficiently implemented in pastoralist settings, locally-appropriate interventions should be devised and tested.

## Supplementary Information

**Additional file 1:** **Table S1.** Number of sampled camels and udder quarters and the number of Group B Streptococcus (GBS)-positive camels and quarters in 20 dairy camel herds in Isiolo county, Kenya. One isolate per camel was selected for whole genome sequencing.

**Additional file 2.** Authors’ original data for 65 GBS-isolates collected from camel milk in Isiolo county, Kenya, February-May, 2017, including accession numbers available at European Nucleotide Archive (ENA).

**Additional file 3: Figure S1.** Maximum likelihood phylogenetic tree of 49 Lac.2 integrase amino-acid sequences extracted from group B Streptococcus genomes from Kenyan camels. Sequences were aligned using MAFFT v7.475 and phylogeny was estimated with PhyML v3.3.20190909. Isolate names are shown. Leaf colours correspond to the insertion site where Lac.2 is integrated (light green: deoD, red: yxdL, blue: hypothetical, purple: ClbS/DfsB family four-helix bundle protein). For 30 isolates, the Lac.2 insertion site could not be determined because their integrases were found at the edge of a contig (dark green leaves).

**Additional file 4: Figure S2.** Organisation of genes of the scpB-lmb mobile transposon as found in group B Streptococcus isolate P4 (sequence type 1) in milk from a camel (Camelus dromedarius) in Kenya. In this transposon, two variants of C5a peptidase gene scpB are present (scpB1 and scpB2) upstream the laminin binding protein gene lmb.

## Data Availability

All data analysed during this study is included in this published article [and its supplementary information files 1–4]. Sequence data generated for this study is available at the European Nucleotide Archive (ENA) under study PRJEB43245 (accession numbers from ERS5810822 to ERS5810886).

## Abbreviations

ACM: Acute clinical mastitis
AMR: Antimicrobial resistance
CC: Clonal complex
CCM: Chronic clinical mastitis
CM: Clinical mastitis
CMT: California mastitis test
GBS: Group B *Streptococcus*
MIC: Minimum inhibitory concentration
Lac.2: Lactose operon
*lmb*: Laminin-binding protein
MALDI-Tof MS: Matrix assisted laser desorption ionization-time of flight mass spectrometry
*scpB*: C5a peptidase
SCM: Subclinical mastitis
ST: Sequence type
SVA: National Veterinary Institute
