# Supplementary material for: Genomic analysis of group B Streptococcus from milk demonstrates the need for improved biosecurity: a cross-sectional study of pastoralist camels in Kenya
Source: BMC Microbiol. 2021 Jul 19;21:217. doi: 10.1186/s12866-021-02228-9 (PMC8287776; doi:10.1186/s12866-021-02228-9)
Supplement: Supplementary file 1 — Additional file 1: Table S1. Number of sampled camels and udder quarters and the number of Group B Streptococcus (GBS)-positive camels and quarters in 20 dairy camel herds in Isiolo county, Kenya. One isolate per camel was selected for whole genome sequencing. [file 12866_2021_2228_MOESM1_ESM.docx]

**Table S1.**

Number of sampled camels and udder quarters and the number of Group B *Streptococcus* (GBS)-positive camels and quarters in 20 dairy camel herds in Isiolo county, Kenya. One isolate per camel was selected for whole genome sequencing.

|  | Number sampled | | GBS-positive | |
| --- | --- | --- | --- | --- |
| Herd-ID | Camels | Quarters | Camels | Quarters |
| A | 10 | 38 | 4 | 12 |
| B | 11 | 44 | 5 | 16 |
| C | 10 | 40 | 2 | 3 |
| D | 10 | 37 | 2 | 5 |
| E | 10 | 40 | 2 | 5 |
| F | 10 | 39 | 1 | 4 |
| G | 11 | 42 | 2 | 5 |
| H | 10 | 39 | 1 | 3 |
| I | 13 | 51 | 5 | 14 |
| J | 10 | 40 | 7 | 18 |
| K | 10 | 38 | 5 | 10 |
| L | 11 | 40 | 1 | 1 |
| M | 11 | 43 | 5 | 11 |
| N | 11 | 44 | 4 | 7 |
| O | 11 | 44 | 4 | 6 |
| P | 10 | 39 | 5 | 8 |
| Q | 7 | 28 | 1 | 2 |
| R | 10 | 38 | 8 | 21 |
| S | 10 | 40 | 0 | 0 |
| T | 11 | 42 | 1 | 3 |
|  |  |  |  |  |
| Total | 207 | 806 | 65 | 154 |
