# Supplementary material for: Genomic analysis of group B Streptococcus from milk demonstrates the need for improved biosecurity: a cross-sectional study of pastoralist camels in Kenya
Source: BMC Microbiol. 2021 Jul 19;21:217. doi: 10.1186/s12866-021-02228-9 (PMC8287776; doi:10.1186/s12866-021-02228-9)

**Figure S1.**

Maximum likelihood phylogenetic tree of 49 Lac.2 integrase amino-acid sequences extracted from group B *Streptococcus* genomes from Kenyan camels. Sequences were aligned using MAFFT v7.475 and phylogeny was estimated with PhyML v3.3.20190909. Isolate names are shown. Leaf colours correspond to the insertion site where Lac.2 is integrated (light green: *deoD*, red: *yxdL*, blue: hypothetical, purple: ClbS/DfsB family four-helix bundle protein). For 30 isolates, the Lac.2 insertion site could not be determined because their integrases were found at the edge of a contig (dark green leaves).


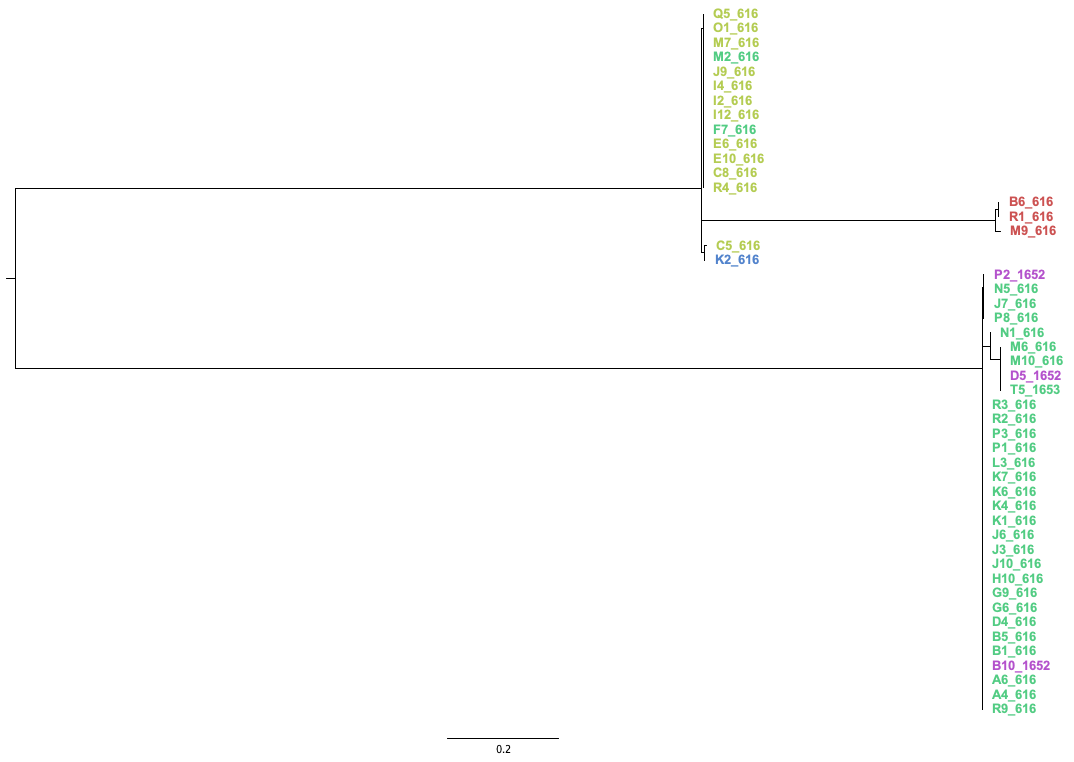

Supplement: Supplementary file 3 — Additional file 3: Figure S1. Maximum likelihood phylogenetic tree of 49 Lac.2 integrase amino-acid sequences extracted from group B Streptococcus genomes from Kenyan camels. Sequences were aligned using MAFFT v7.475 and phylogeny was estimated with PhyML v3.3.20190909. Isolate names are shown. Leaf colours correspond to the insertion site where Lac.2 is integrated (light green: deoD, red: yxdL, blue: hypothetical, purple: ClbS/DfsB family four-helix bundle protein). For 30 isolates, the Lac.2 insertion site could not be determined because their integrases were found at the edge of a contig (dark green leaves). [file 12866_2021_2228_MOESM3_ESM.docx]
