# Supplementary material for: Genomic analysis of group B Streptococcus from milk demonstrates the need for improved biosecurity: a cross-sectional study of pastoralist camels in Kenya
Source: BMC Microbiol. 2021 Jul 19;21:217. doi: 10.1186/s12866-021-02228-9 (PMC8287776; doi:10.1186/s12866-021-02228-9)

**Figure S2.** Organisation of genes of the *scpB-lmb* mobile transposon as found in group B *Streptococcus* isolate P4 (sequence type 1) in milk from a camel (*Camelus dromedarius*) in Kenya. In this transposon, two variants of C5a peptidase gen *scpB* are present (*scpB1* and *scpB2*) upstream the laminin binding protein gene *lmb*.


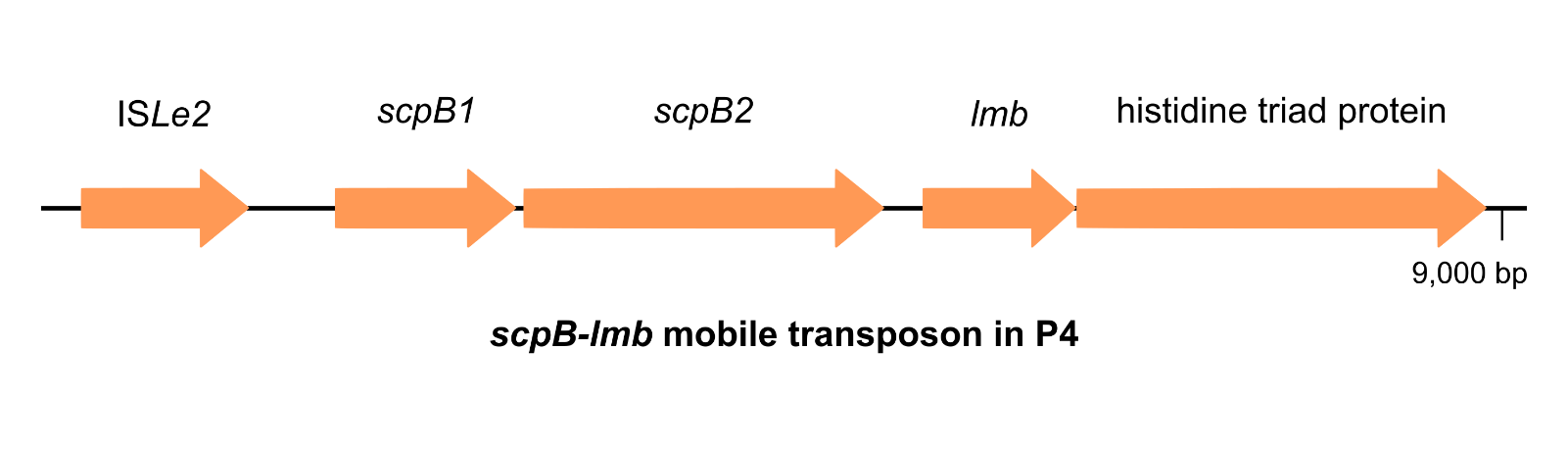

Supplement: Supplementary file 4 — Additional file 4: Figure S2. Organisation of genes of the scpB-lmb mobile transposon as found in group B Streptococcus isolate P4 (sequence type 1) in milk from a camel (Camelus dromedarius) in Kenya. In this transposon, two variants of C5a peptidase gene scpB are present (scpB1 and scpB2) upstream the laminin binding protein gene lmb. [file 12866_2021_2228_MOESM4_ESM.docx]
